# Cell-Autonomous and Non-Cell-Autonomous Roles for *Irf6* during Development of the Tongue

**DOI:** 10.1371/journal.pone.0056270

**Published:** 2013-02-22

**Authors:** Steven Goudy, Peggi Angel, Britni Jacobs, Cynthia Hill, Veronica Mainini, Arianna L. Smith, Youssef A. Kousa, Richard Caprioli, Lawrence S. Prince, Scott Baldwin, Brian C. Schutte

**Affiliations:** 1 Department of Otolaryngology, Vanderbilt University, Nashville, Tennessee, United States of America; 2 Department of Imaging/Biophysics, Vanderbilt University, Nashville, Tennessee, United States of America; 3 Department of Health Sciences, Universita degli Studi Di Milano-Bicocca, Milan, Italy; 4 Genetics PhD Program, Michigan State University, East Lansing, Michigan, United States of America; 5 Department of Biochemistry and Molecular Biology, Michigan State University, East Lansing, Michigan, United States of America; 6 Division of Neonatology, Vanderbilt University, Nashville, Tennessee, United States of America; 7 Department of Cell and Developmental Biology, Vanderbilt University, Nashville, Tennessee, United States of America; 8 Department of Pediatrics, Vanderbilt University, Nashville, Tennessee, United States of America; 9 Division of Cardiology, Vanderbilt University, Nashville, Tennessee, United States of America; 10 Departments of Microbiology and Molecular Genetics and Pediatrics and Human Development, Michigan State University, East Lansing, Michigan, United States of America; University of Massachusetts Medical School, United States of America

## Abstract

Interferon regulatory factor 6 (*IRF6*) encodes a highly conserved helix-turn-helix DNA binding protein and is a member of the interferon regulatory family of DNA transcription factors. Mutations in *IRF6* lead to isolated and syndromic forms of cleft lip and palate, most notably Van der Woude syndrome (VWS) and Popliteal Ptyerigium Syndrome (PPS). Mice lacking both copies of *Irf6* have severe limb, skin, palatal and esophageal abnormalities, due to significantly altered and delayed epithelial development. However, a recent report showed that *MCS9.7*, an enhancer near *Irf6*, is active in the tongue, suggesting that *Irf6* may also be expressed in the tongue. Indeed, we detected Irf6 staining in the mesoderm-derived muscle during development of the tongue. Dual labeling experiments demonstrated that Irf6 was expressed only in the *Myf5*+ cell lineage, which originates from the segmental paraxial mesoderm and gives rise to the muscles of the tongue. Fate mapping of the segmental paraxial mesoderm cells revealed a cell-autonomous *Irf6* function with reduced and poorly organized *Myf5*+ cell lineage in the tongue. Molecular analyses showed that the *Irf6*−/− embryos had aberrant cytoskeletal formation of the segmental paraxial mesoderm in the tongue. Fate mapping of the cranial neural crest cells revealed non-cell-autonomous *Irf6* function with the loss of the inter-molar eminence. Loss of *Irf6* function altered *Bmp2*, *Bmp4*, *Shh*, and *Fgf10* signaling suggesting that these genes are involved in Irf6 signaling. Based on these data, *Irf6* plays important cell-autonomous and non-cell-autonomous roles in muscular differentiation and cytoskeletal formation in the tongue.

## Introduction

Interferon regulatory factor 6 (*IRF6*) encodes a highly conserved winged-helix DNA binding protein and is a member of the interferon regulatory family of DNA transcription factors. IRF6 is highly expressed in epithelial cells and mutations in *IRF6* are responsible for Van der Woude (VWS) and popliteal pterygium syndrome (PPS), two autosomal dominant forms of cleft lip and palate. Mutations in *IRF6* lead to multiple associated findings, from lower lip pits in VWS patients to webbing of the limbs, syngnathia, ankyloblepharon, and urogenital abnormalities in PPS patients [1]. In mice with mutations in the *Irf6* gene, a wide array of similar anomalies seen in PPS patients were observed such as webbing of the limbs, syngnathia, and esophageal adhesion [2], [3]. In examining the cleft palate phenotype of these mice, there were severe adhesions throughout the oral cavity epithelium, preventing normal palate elevation and apposition. Evaluation of the cutaneous epithelium in the *Irf6*−/− mice revealed aberrant development characterized by poor organization and lack of differentiation. To date all of the investigation on *Irf6* mice has focused on the epithelial phenotype, but the requirement of *Irf6* during mesenchymal development is unknown.

There have not been previous reports of *Irf6* function in the tongue, however previous authors demonstrated *Irf6* expression in the tongue using *in situ* hybridization [4], [5]. In addition, a recent study showed that the enhancer element, *MCS9.7* was highly active in the developing tongue and limb musculature [6]. *MCS9.7* is a multi-species conserved sequence that is located 9.7 kb upstream of the transcriptional start site for *IRF6*. It was originally identified as a likely enhancer for *Irf6* using a transient transgenic embryo assay [7]. The recent study used stable transgenic lines to demonstrate that *MCS9.7* enhancer activity replicated endogenous *Irf6* expression in most tissues. Moreover, due to the enhanced sensitivity of this transgenic system, new candidate regions of *Irf6* expression were detected including the developing somites and in a subpopulation of the intrinsic tongue mesoderm in the same location as the transversal muscles of the tongue [6]. These data are consistent with our hypothesis that *Irf6* is necessary for lingual mesenchymal development.

Lingual development occurs as a migration of segmental paraxial mesoderm from the area adjacent to the dorsal neural tube to invest into the floor of the mouth where the tongue will form (reviewed by Yamane 2005) [8]. These mesodermal cells migrate and proliferate via induction by *Shh* and are inhibited by *Bmp*s. Once segmental paraxial mesodermal (SPM) cells have migrated into the tongue, they undergo determination as myoblasts, differentiation into myotubes and maturation into myofibers. The cell populations that comprise the tongue are derived from both cranial neural crest (CNC) that give rise to tongue connective tissue and SPM cells that give rise to the tongue musculature. Differentiation of the SPM cells into myocytes begins at E13 and is complete by E15. The markers of tongue muscle differentiation include *Myf5*, myogenin, and myosin heavy chain. At birth the tongue musculature has completed maturation and is fully functional (reviewed by Yamane) [8].

In this paper we hypothesized that *Irf6* is not only required for epithelial development, but that *Irf6* also guides lingual mesodermal and mesenchymal development using cell-autonomous and non-cell-autonomous mechanisms, respectively. To determine the role(s) that *Irf6* plays during tongue development, we evaluated lineage tracing of the tongue cells (*Wnt1-Cre* for CNC cells and *Myf5*-*Cre* for SPM), measured the effect of *Irf6* loss on the cell cycle, and evaluated possible mediators and targets of *Irf6* signaling in the tongue.

## Materials and Methods

### Murine Crosses and Genotyping

*Irf6*+/− mice were maintained on C57BL/6J background and have been previously described [2]. The *Irf6*+/− mice were mated with *Wnt1-Cre* and *Myf5*-*Cre* mice that also contained the *ROSA26* reporter strain (*R26R*), all of which have been previously described, to create *Irf6*−/−;*Wnt1-Cre*;*R26R* and *Irf6*−/−;*Myf5*-Cre;*R26R* mice [9], [10]. Embryos were staged considering that the day a plug was identified was considered day E 0.5. Each embryo was washed in 1x cold phosphate-buffered saline and then fixed with 4% paraformaldehyde for up to 4 hours and then processed for immunohistochemistry. Genotyping was performed on DNA from yolk sac or tail samples for polymerase chain reaction as described in the above referenced papers. This study was performed under a Vanderbilt IACUC approved protocol to study *Irf6* and all procedures were done in accordance with Vanderbilt IACUC guidelines. Three separate biologic specimens for each genotype of control and *Irf6*−/− mice were sectioned, stained and examined for specific immuno-staining as indicated below.

### Immuno-staining

Florescence staining was performed on serial coronal sectioning of OCT embedded tissue. Eight micrometer thick sections were thawed and rehydrated in phosphate buffered saline. The sections were treated with 0.1% Tween and blocked with 10% donkey serum. The following antibodies were used: 1^o = ^ Irf6 antimouse/rabbit polycloncal (Fakhouri et al., 2012), Beta-Galactosidase anti-mouse chicken polyclonal (Abcam), Phalloidin-Alexa conjugate anti-mouse goat monoclonal (Life), CD44 anti-mouse rabbit polyclonal (Abcam), MF-20 anti-mouse chicken monoclonal (gift from David Bader), Beta Thymosin 4 anti-mouse rabbit polyclonal (Abcam), Beta Thymosin 10 anti-mouse rabbit polyclonal (Abcam). 2^o^ = Cy3 AntiGoat/Donkey, Alexa 488 AntiRat/Goat Polyclonal. Hard Mount with Dapi® was used to counterstain (Vectastain). Imaging was performed on a Nikon E800 microscope; and images were obtained with SPOT® software. Phalloidin stained sections were imaged by laser scanning microscopy using a Leica SPE-2 confocal system and a 63x/1.4N.A. objective. Three-dimensional series of images were obtained in z for both control and mutant samples spanning 4 um in depth and spaced 0.13 um apart. Maximal projection images of all sections are shown.

### PhosphoHistone-3 Immunohistochemistry

7 µm paraffin coronal sections were dewaxed and rehydrated prior to unmasking with a boil in 10 mM Tris–pH 10.0. The sections were quenched in 3% hydrogen peroxide, and then blocked with 5% goat serum. The sections were incubated with 1^o^ antibody - Phospho Histone 3 (9701L, Cell Signaling Technology), and stained using ABC Kit® according to the standard protocol (Vector). DAB reagent was carefully added to the sections and watched under Nikon E800 for development. The sections were immediately washed and counterstained with Hematoxylin. Brightfield microscopy (Nikon E800) was used for cell counting in the palate shelves. Three separate biologic specimens from control and *Irf6*−/− mice were examined in triplicate for proliferation. The results were compared using a paired students *t*-test to establish significance. P-values of <0.05 were considered significant.

### Hyaluronic Acid Histochemistry

Standard paraffin sections were dewaxed and rehydrated prior to unmasking with dilute bovine testicular hyaluronidase. The sections were washed; then blocked with 3% acetic acid. Alcian Blue (pH 2.5) was utilized for staining. The sections were washed prior to counterstaining with nuclear fast red. Three separate control and *Irf6*−/− specimens were sectioned, stained with Alcian Blue and examined at E14.5.

### Quantitative PCR

To determine changes in gene expression, we used qRT-PCR as previously described [11]. Total RNA was isolated using the TRIzol reagent (Invitrogen). cDNA was generated from 1 µg total RNA using oligo-dT primers and Superscript III polymerase (Invitrogen). Primer pairs are shown below. Real-time PCR analysis was done with iQ SYBR green supermix (Bio-Rad) in the Bio-Rad iCycler for 40 cycles. The expression levels are calculated using the ΔΔC_T_ method. The threshold cycle (C_T_) represents the PCR cycle at which an increase of the reporter fluorescence above the baseline is first detected. The fold change in expression levels, R, is calculated as follows: R = 2^−ΔΔCT^ (where R  = 2 ^(ΔCT treated-ΔCT control)^) to normalize the abundance of all transcripts to the level of *GAPDH* RNA expression. Three separate biologic specimens of control and *Irf6*−/− tongue tissues were analyzed in triplicate at E14.5. The quantitative PCR results were compared using a paired students *t*-test to establish significance. P-values of <0.05 were considered significant. The primers used for sense (5′**→**3′)/anti-sense (5′**→**3″) were: *GAPDH* atgacaatgaatacggctacag, tctcttgctcagtgtccttg; *Irf6* catgccattt-atgccatcag, aaaaggcggctgcttctcta; *BMP2* ttatcaggacatggttgtggag, gggaaatatta-aagtgtcagctgg; *BMP4* gtagtgccattcggagcg, atcagcattcggttaccagg; *Shh* tccaaag-ctcacatccactg, cgtaagtccttcaccagcttg; *Fgf10* gtcctggagataacatcagtgg, tctct-ttcagcttacagtcgttg.

### Sample Preparation for MALDI Imaging Mass Spectrometry

Fresh frozen E13.5 heads were embedded in OCT, cryosectioned to 12 µm thickness, thaw-mounted onto indium-tin oxide (ITO)-coated glass slides and vacuum dehydrated for 2 hours. Sections were washed sequentially with 70, 90 and 95%ethanol for 30 s, [12] allowed to dry in a vacuum desiccator 1 h and stored at -80°C. Prior to imaging mass spectrometry, sections were washed 1 min with Carnoy’s solution (Ethanol/Chloroform/Acetic Acid = 6/3/1), 30 seconds in distilled water, and 30 seconds in 100% ethanol. Sections were coated with sinapinic acid (SA) MALDI matrix by sublimation and recrystallized as described previously. [13] AfterIMS, MALDI matrix was removed by washing for 30 seconds in methanol and sections were stained for optical imaging with hematoxylin and eosin. Methanol, ethanol and chloroform were purchased from VWR (Radnor, PA, USA). Acetic acid was purchased from Fisher Scientific (Pittsburgh, PA, USA). Sinapinic acid was purchased from Acros-Organics (Morris Plains, NJ, USA) and recrystallized in-house to remove impurities.

### MALDI-TOF Data Acquisition

Imaging data were acquired using a linear mode MALDI-TOF mass spectrometer (Autoflex Speed, Bruker Daltonics, Bremen,Germany) in positive ion mode with a range extending from 2000 to 20,000* m/z*. The source was set to an accelerating voltage of 19.5 kV with an extraction voltage of 18.2 kV, a lens voltage of 6.0 kV and a pulsed ion extraction time of 350 ns. Ionization was performed with a Smartbeam2 Nd:YAG laser operated at a 1 kHz repetition rate, focused to 94% within 100% of the focus range. The laser attenuator offset was adjusted to 60% and laser fluency was operated at 70%. Imaging data were acquired by summing up 150 shots per array position with each array position collected at 50 µm intervals. External calibration was performed using bovine insulin ([M+H] = *m/z* 5734.5), equine cytochrome C ([M+H] = *m/z* 12361.2), equine apomyoglobin ([M+H] = *m/z* 16952.5), bovine trypsinogen([M+H] = *m/z* 23982.0).

### MALDI-TOF Image Processing and Data Analysis

The imaging data were normalized to total ion current and visualized using the FlexImaging software package (version 3.0; Bruker Daltonics). ClinProTools 2.2 software was used for the statistical analysis and multiple spectral comparison (WT, n = 4; *Irf6* (−/−), n = 4; total spectra from each population 1,000). The mass range of interest was restricted to *m/z* 2000 to 15000. Spectra were internally calibrated on common peaks to 1000 parts per million (ppm) and automatically normalized to total ion current. The spectra were baseline subtracted using the ’Top Hat’ algorithm with a minimum baseline width of 10% and smoothed using the Savitsky- Golay smoothing filter set at a 2 Da width over five cycles. Peak calculation was based on peak intensity and was performed with a signal-to-noise (S/N) ratio of 5 on the total average spectrum of each class. The peak intensities considered statistically significant between classes were based on a combination of ANOVA test (p-value <0.05) and Receiver Operating Curve (ROC) analysis (AUC >0.8).

### Protein Identification

E13.5 tongue tissue was homogenized by 5 minutes sonication in 20% acetonitrile with HALT protease. Homogenate was separated by reverse phase column (C8). Solvent A was 0.1% formic acid; solvent B was acetonitrile in 0.1% formic acid. Proteins were eluted at a 0.75% B/minute gradient into an LTQ Orbitrap Velos (Thermo Scientific). Data dependent fragmentation was performed on selected precursors, alternating collision-induced dissociation (CID) with electron transfer dissociation (ETD). After each survey scan, the 10 most intense precursors with an ion threshold of ≥1000 within an isolation width of 2.5 Da were selected for subsequent fragmentation. Normalized collision energy was set to 35% for both CID and ETD. Activation time for CID was 10 ms and 100 ms for ETD. Spectra were manually interpreted, comparing sequences by blast search (Uniprot.org).

## Results

### *Irf6* is Expressed in Segmental Paraxial Mesoderm During Lingual Development

**Figure 1:**
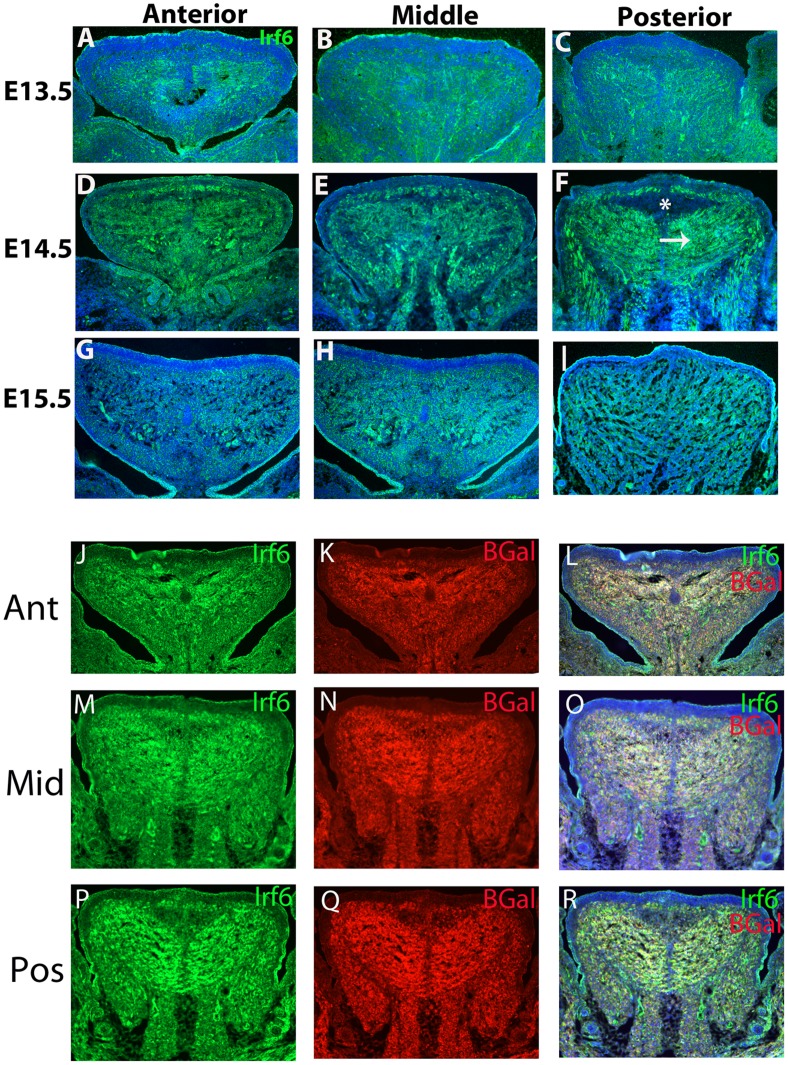
Expression of *Irf6* in Tongue Occurs in *Myf5*+ Cell Lineage. Coronal sections through the anterior, middle and posterior tongue during critical stages of tongue differentiation performed in triplicate (E13.5–E15.5). At E13.5 there was moderate *Irf6* expression in the tongue giving a striated appearance representing the transversal muscle extending from the midline (A, B, C). At E14.5 there was intense *Irf6* staining throughout the tongue (D, E, F) with more prominent striations of the transversal muscle extending from the midline (white arrow) and an *Irf6*-free zone in the inter-molar eminence (white asterisk). At E15.5 there was a reduction in *Irf6* signaling throughout the tongue (G, H, I). Lineage tracing of segmental paraxial mesoderm was performed using *Myf5-Cre;R26R* mice. *Irf6* staining occurs in the striations of the transversal muscle of the tongue tissue (J, M, P) and detection of B-Galactosidase activity was found in the same region of the tongue of *Myf5-Cre;R262R* mice (K, N, Q). Overlay of these images demonstrated that *Irf6* staining occurred primarily in the *Myf5*+ cell lineage (L, O, R).

Previous studies showed that Irf6 was expressed primarily in the epithelium of the tongue. However, cell lineage tracing of *MCS9.7*, an enhancer element whose activity replicates most of the endogenous Irf6 pattern of expression, suggested that Irf6 may also be expressed in mesodermal tissues in the developing tongue [6]. Using a previously characterized antibody for Irf6 (Fakhouri et al., 2012), we observed strong expression of Irf6 in the lingual epithelium, as expected, but also in the lingual mesoderm from E13.5 to E15.5 (Figure 1). Irf6 was detected throughout the anterior, middle and posterior aspects of the lingual mesoderm of control samples in triplicate, although not all lingual cells expressed Irf6. The strongest expression of Irf6 in the tongue was at E14.5 (Figure 1 D, E, F), compared to E13.5 (Figure 1 A, B, C) and E15.5 (Figure 1 G, H, I). Expression of Irf6 in the lingual mesoderm occurred along striations of cells extending from the midline lingual raphe representing the transversal muscle (best seen in Figure 1 F, white arrow). However, there was a bulge of cells in the posterior E14.5 tongue that was free of Irf6 expression. This bulge was located at the inter-molar eminence (Figure 1 F, asterisk). We conclude that Irf6 was expressed in lingual mesodermal cells, as well as epithelial cells, as previously suggested by the enhancer activity of *MCS9.7*.

The cells of the tongue originate from two sources, cranial neural crest (CNC) cells and segmental paraxial mesoderm (SPM) cells. The SPM cells contribute to the lingual muscular formation whereas the CNC cells contribute to the supporting stromal cells of the connective tissue [14]. The distribution of the SPM in the lingual mesoderm appeared very similar to the pattern of expression for *Irf6*. To determine whether *Irf6* is expressed in the SPM, we used the *Myf5-Cre;R26R* transgenes to lineage trace cells from the segmental paraxial mesoderm [14]. We performed dual immuno-staining for Irf6 and beta-galactosidase (Bgal) in triplicate on 3 separate biologic specimens of coronal sections of embryonic heads in the *Myf5-Cre;R26R* transgenic strain (Figure 1 L, O, R). We observed nearly complete overlap of the Irf6 and Bgal staining in the anterior, middle and posterior tongue mesoderm in the embryos that carried the *Myf5-Cre* transgene. These data further support that most Irf6 was produced in the lingual mesoderm.

### Aberrant Segmental Paraxial Mesodermal and Neural Crest Development During Lingual Formation in *Irf6*−/− Mice

**Figure 2:**
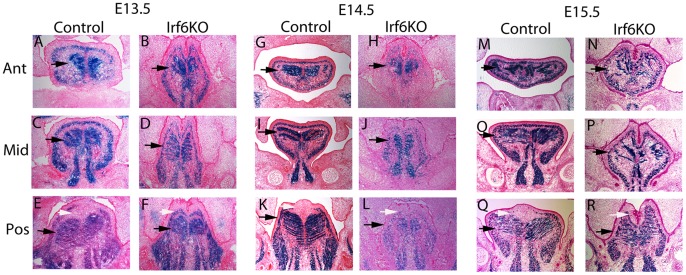
Lineage tracing SPM using *Myf5-Cre;R26R* in *Irf6*−/− Embryos. Coronal sections through anterior, middle and posterior tongue of control and *Irf6*−/− mice at E13.5, E14.5, and E15.5 performed in triplicate. In the E13.5 control tongues there was a strong contribution of the SPM (*Myf5*+) to the tongue (blue cells in A, C, E) with striations extending from the midline representing the transversal muscle (black arrow). In the *Irf6*−/− tongues there was reduced SPM (blue cells in B, D, F) with reduced and poorly organized striations of transversal muscle (black arrow). At E14.5 there was increased SPM in the control tongues (blue cells in G, I, K) and more striations of the transversal muscle (black arrow). In the E14.5 *Irf6*−/− tongue there was reduced SPM (blue cells in H, J, L) and reduction in striations of the transversal muscle and poor organization (black arrows). In the E15.5 control tongue there was primarily SPM (blue cells in M, O, Q) with increasing striations of the transversal muscle (black arrows). In the E15.5 *Irf6*−/− tongue there was a significant reduction of SPM (blue cells in N, P, R) with reduced striations of the transversal muscle and poor organization (black arrows). The inter-molar eminence was not populated by SPM and was absent in the *Irf6*−/− tongue (white arrows in E, K, Q).

*Irf6* is required for regulation of proliferation and differentiation during epithelial development [2], [3]. To test whether *Irf6* has a similar role in the lingual mesodermal cell proliferation and differentiation, we performed the same lineage tracing experiments as above, except in embryos that lacked *Irf6* in triplicate [9], [10]. Using the SPM lineage trace, we observed three main patterns (Figure 2). First, in the wild type mice, there were two broad areas of staining, a ring of staining below the surface of the tongue representing the superior longitudinal muscle and two sets of horizontal striations that flanked the midline of the tongue where the transversal muscle forms (Figure 2). The ring of staining was continuous, except in the posterior region of the tongue where there was a break at the midline that co-localized with the inter-molar eminence of the tongue (Figure 2K, white arrow). The second pattern was that the horizontal striations of the transversal muscle in the wild type embryos vary by age and location. As the control embryo ages, the horizontal striations of the transversal muscles became more defined and more numerous. The relative number of horizontal striations of the transversal muscle also increased in sections taken from the anterior to the posterior regions of the tongue. Third, in the *Irf6*−/− embryos, the ring of staining of the superior longitudinal muscle in the posterior tongue was not discontinuous (i.e. was continuous), and the inter-molar eminence was not visible. Also, while the horizontal striations of the transversal muscle were present in the mutant embryos, they appeared less organized, and they were reduced in the base of the tongue. In sum, the absence of *Irf6* affected SPM by reducing the relative number of horizontal SPM striations of the transversal muscle, especially at the base of the tongue, and by reducing the organization of all the horizontal striations of the transversal muscle. Since *Irf6* was expressed in the SPM and is a putative transcription factor, we suggest that these effects were cell autonomous. Of note, the cells in the posterior inter-molar eminence of the tongue did not originate from SPM. Thus, the apparent loss of the inter-molar eminence in embryos that lack *Irf6* was consistent with a non-cell autonomous effect.

To determine if Irf6 was expressed in the CNC, we co-labeled Irf6 and B-gal activity in *Wnt1-Cre;R26R* mice in triplicate (Figure S1). There was minimal evidence to suggest that Irf6 was expressed in the CNC cell lineage (Figure S1 C, F, I). To test the effect of *Irf6* loss on the cranial neural crest cells, we generated embryos that lack Irf6 and carry the *Wnt1-Cre;R26R* transgenes. We observed three main patterns of B-gal activity. First, in the E13.5 wild type embryos, the majority of the tongue was blue suggesting that most of the tongue mesenchymal cells were derived from cranial neural crest cells (Figure S2). However, as expected from the study of *Myf5-Cre;R26R* embryos, there are striations of unstained (pink) cells representing the transversal muscle that extend out from the midline lingual raphe, and a ring of unstained cells that parallel the surface of the tongue representing the superior longitudinal muscle (Figure S2 A, C, E). In the posterior region of the tongue, the ring of unstained cells of the superior longitudinal muscle was discontinuous, and the inter-molar eminence stained blue suggesting that the inter-molar eminence was derived from cranial neural crest. Second, in the E14.5 and E15.5 wild type embryos, the fraction of CNC-derived cells (blue) appeared to decrease especially in the mid- and posterior regions (Figure S2 K, Q). These data suggest that the segmental paraxial mesoderm contributed more heavily to growth of the tongue in the latter stages of development, during lingual myogenic determination and differentiation. Third, in the *Irf6*−/− embryos, the decrease in CNC-derived mesenchyme seen in the control mice appeared to be attenuated, especially in the mid- and posterior sections of the E14.5 and E15.5 embryos (white arrows). The results with the CNC cell lineage tracer were consistent with the results with the lineage tracing of the SPM. Both showed that *Irf6* was required for development of the inter-molar eminence, and since *Irf6* was expressed solely in the SPM and epithelium, then we concluded that the mechanism for this *Irf6* function was non-cell-autonomous.

### Altered Proliferation in the Tongue of *Irf6*−/− Embryos

**Figure 3:**
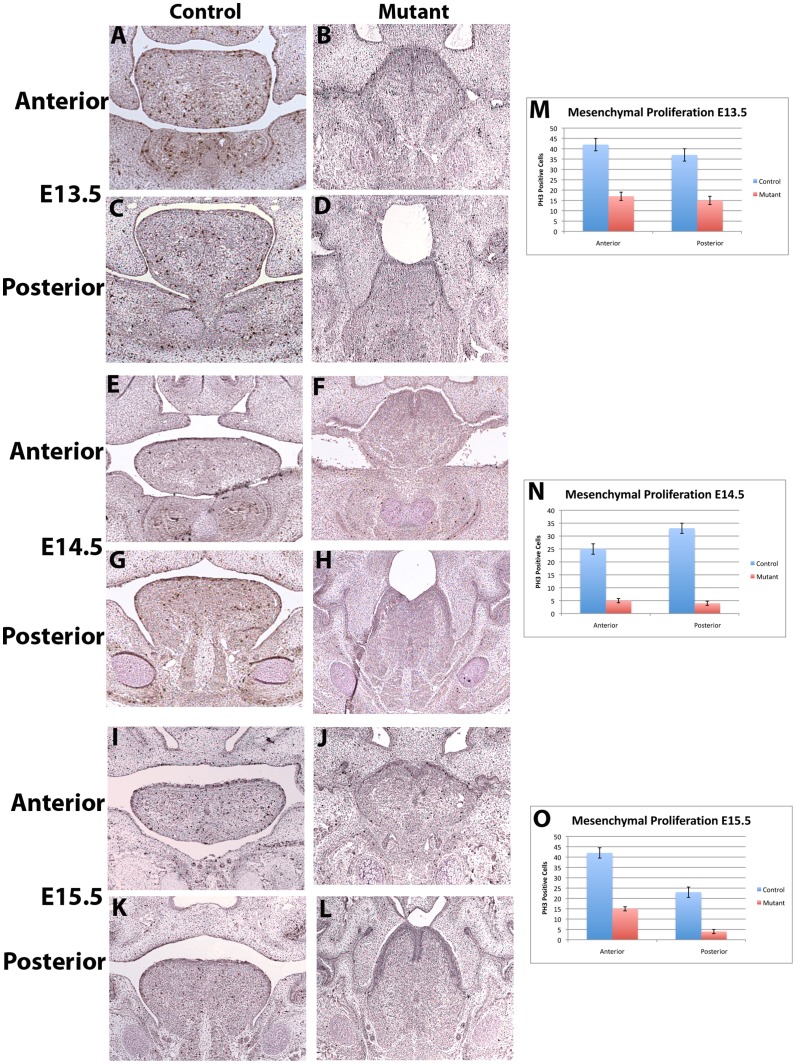
Reduced Cellular Proliferation in the *Irf6*−/− Tongue. Coronal sections through anterior, middle and posterior tongue of control and *Irf6*−/− mice at E13.5, E14.5, and E15.5 performed in triplicate. Using phosphohistone H3 (PH3) to label proliferative cells we identified PH3 reductions in E13.5 *Irf6*−/− anterior and posterior tongue (B, D) compared to control (A, C) that was significant (M). In the E14.5 mice tongues there was a dramatic decrease in PH3 labeled cells in the control tongue (E, G) compared to *Irf6*−/− tongues (F, H) that was significant (N). At E15.5 there continued to be reduced cellular proliferation in the *Irf6*−/− tongue (J, L) compared to controls (I, K) that was significant (Q). Statistical significance was determined using a student t test and P<0.05 used to determined significance (*).

Based on the profound effect that *Irf6* deletion exhibits on the SPM cell populations in the tongue, we chose to examine alterations in proliferation. Using phosphohistone H3 (Figure 3) and BrdU (data not shown) staining in triplicate, we identified a statistically significant reduction in cellular proliferation in the anterior and posterior tongue of the *Irf6*−/− mice at E13.5 (Figure 3 B, D, M). At E14.5 the reduction in proliferation in the *Irf6*−/− mice was more severe (Figure 3 F, H, N) than at E13.5 and may explain the reduced *Myf5*+ cells seen at E14.5 (Figure 2 H, J, L). The reduction in proliferation at E14.5 in the tongue was also more noticeable in the posterior region of the tongue of the *Irf6*−/− mice, compared to the anterior tongue. This was consistent with the observation that the SPM cells contributed more to the posterior of the tongue than CNC cells. At E15.5 there continued to be reduction of proliferation in the tongue (Figure 3 J, L, O). These data demonstrated that *Irf6* had effects on cell cycle during tongue development, and the decrease in proliferation contributed to the decrease in SPM in the tongue mesoderm. Reduction in cellular proliferation in the mutant embryos occurred concomitantly with the reduction of *Myf5*+ cells during lingual development. Reduction of cellular proliferation in CNC and SPM was seen using co-labeling phosphohistone H3 (PH3) and *Myf5-cre;R26R* transgenic control and *Irf6*−/− mice (Figure S3). TUNEL and caspase 3 staining of control and *Irf6*−/− tongues at E13.5, E14.5 and E15.5 tongues did not reveal any statistical difference in apoptosis (data not shown). These data are most consistent with a reduction in proliferation of SPM and CNC cells in a cell-autonomous and a non-cell-autonomous mechanism in the SPM and CNC, respectively.

### Actin Cytoskeletal Defects in the Tongue of Irf6−/− Embryos

**Figure 4:**
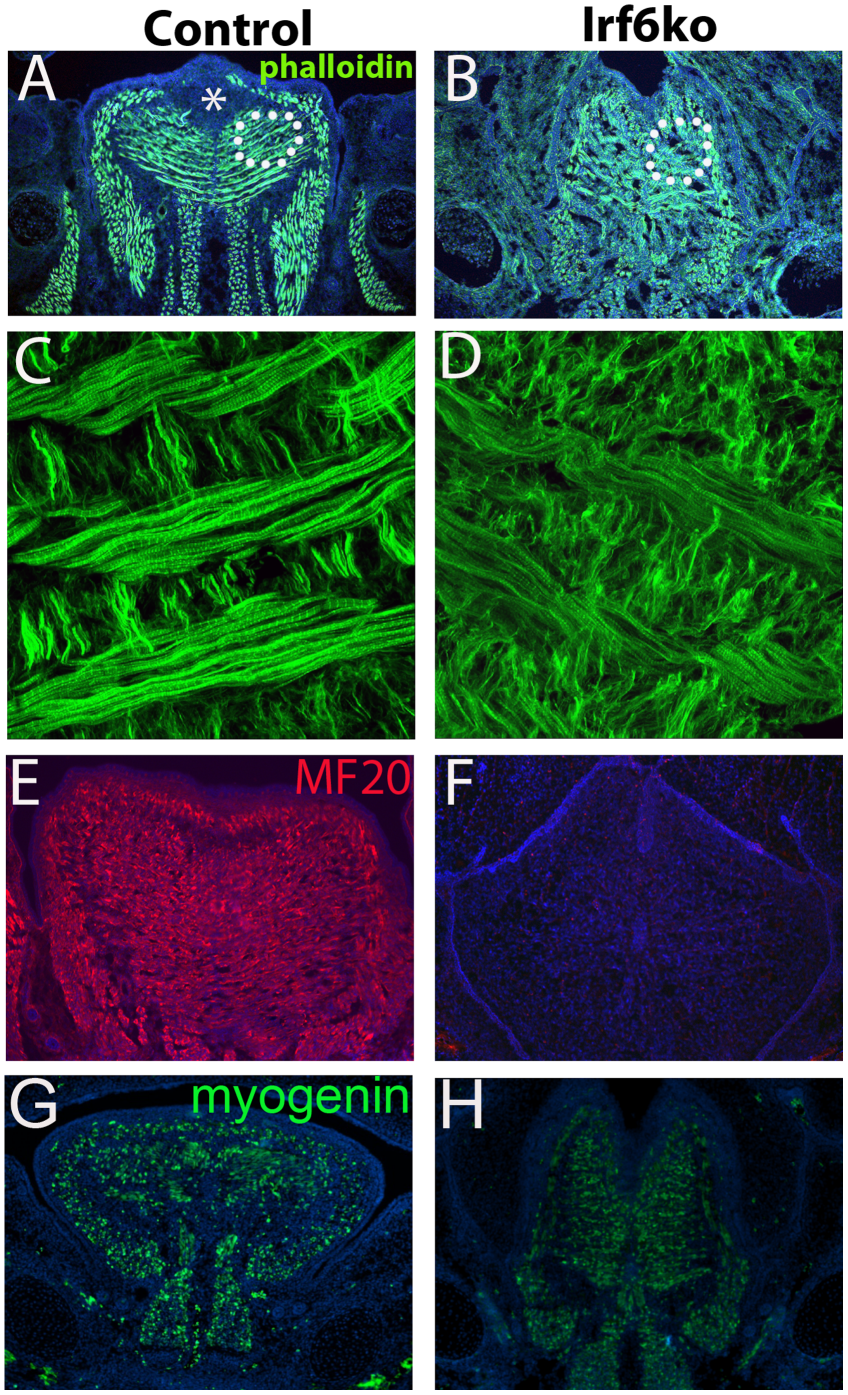
Altered Actin Cytoskeleton in *Irf6*−/− Tongue. Coronal sections of E14.5 control and *Irf6*−/− tongue stained with Phalloidin to detect F-actin performed in triplicate. In the *Irf6*−/− tongue there was reduced Phalloidin staining and poor organization of the stained fibers (B) compared to controls (A). Using confocal imaging on phalloidin-stained images of similar areas of the tongue (denoted by circle in images A, B) we identified reduced actin filament length and poor organization of actin fibers in the *Irf6*−/− fibers (D) compared to controls (C). Myosin heavy chain expression was greatly reduced in E15.5 *Irf6*−/− tongue using MF-20 staining (F) compared to controls (E). Myogenin was not effected in *Irf6*−/− tongue (H) compared to controls (G) * denotes inter-molar eminence.

To examine the effects of *Irf6* loss in the tongue SPM differentiation, we evaluated actin and myosin expression. During skeletal muscle development, *Myf5* is required for myocyte determination and differentiation leading to the formation of actin and myosin in the myofibril [15]. We used phalloidin staining to detect polymerized actin. In triplicate control embryos, the phalloidin stain traced the horizontal fibers of the transversal muscle, as well as the superior longitudinal muscle fibers that ring the surface of the tongue (Figure 4A). However, in triplicate mutant embryos, the actin cytoskeleton was poorly organized (Figure 4 B). Confocal images of coronal sections of the tongue from *Irf6*−/− embryos revealed shorter actin filaments, and poor organization of the polymerized actin filaments in triplicate (Figure 4 D). Myosin heavy chain staining was greatly reduced in E15.5 *Irf6*−/− mice in triplicate (Figure 4 F). We did not observe an obvious difference in staining with myogenin in triplicate (Figure 4 G, H), a key differentiation factor for muscle [16]. These data demonstrated that *Irf6* was required for actin-myosin cytoskeletal polymerization and differentiation of tongue myofibrils.

### Increased B Thymosin 4 and 10 in the *Irf6*−/− Tongue Tissue

**Figure 5:**
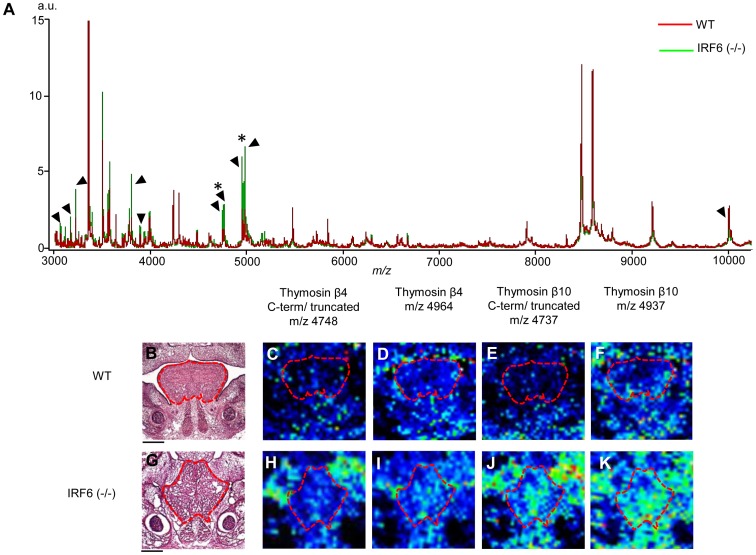
*Beta Thymosin 4* and *Beta Thymosin 10* were Increased in *Irf6*−/− Tongue. *In situ* proteomic analysis of the E14.5 control and *Irf6*−/− tongue in triplicate identified significantly increased mass/charge(m/z) ratios in *Irf6*−/− mice tongues (A, black arrows) at several locations including 4964 (I) and 4937 (K). Protein identification revealed these m/z peaks to be *Beta Thymosin 4* (*BT4*) and *Beta Thymosin 10* (*BT10*). Not only were *BT4* and *BT10* increased in the *Irf6*−/− tongues, so were the C-terminal truncated forms (H) and (J), respectively. Arrows denote significant change in expression (P<0.5) and * denotes m/z areas for BT4 and BT10.

To evaluate changes in steady state levels of proteins involved in the *Irf6* signaling pathway, we analyzed *in situ* protein expression within the tongue of control and *Irf6*−/− mice at E14.5 using MALDI imaging mass spectrometry in triplicate. We detected 18 differentially regulated peptide species associated with loss of *Irf6* (Figure 5 A). We identified two peptide species that showed a large increase in the mutant tissue (Figure 5A, asterisks); Thymosin B4 (BT4) (Figure 5 D, I) and Thymosin B10 (BT10) (Figure 5 F, K). BT4 and BT10 also had a MALDI couplet that represented the C-terminal truncated form of these peptides (Figure 5 C, H and E, J). We confirmed the increase in BT4 and BT10 in the lingual mesenchyme by immuno-staining (Figure S4). These data suggested that *Irf6* repressed BT4 and BT10 expression in the lingual mesenchyme.

### Reduced Hyaluronic Acid and CD 44 in Inter-molar Eminence of *Irf6*−/− Mice

**Figure 6:**
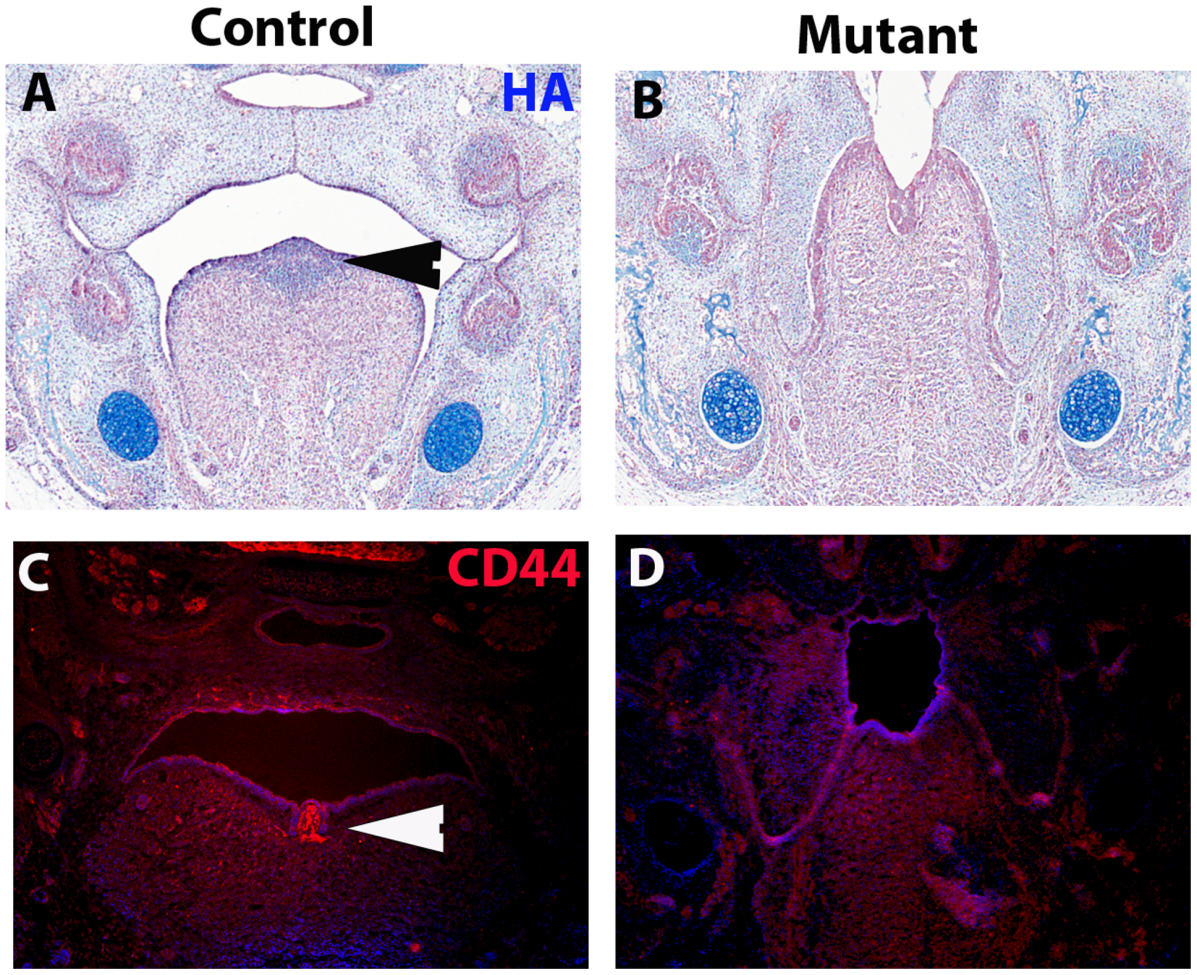
Reduced Hyaluronic Acid and CD44 staining in the *Irf6*−/− Tongue. The inter-molar eminence had Wnt1+ cells however did not contain *Irf6* expression or *Myf5*+ cells. Hyaluronic Acid (HA) staining done at E14.5 showed that the intra-molar eminence was comprised of HA (blue staining in A) and was absent in the *Irf6*−/− mice (B) in triplicate samples. Furthermore, CD44, a receptor for HA signaling, was reduced (red staining) in the *Irf6*−/− posterior tongue (D) compared to controls (C) in triplicate samples.

In triplicate control embryos we observed that the inter-molar eminence disrupted superior longitudinal muscle layer of SPM cells (Figure 2) and was comprised of CNC cells (Figure S2). In triplicate *Irf6*−/− embryos, the inter-molar eminence was absent and the layer of superior longitudinal muscle SPM cells was continuous (Figure 2). The inter-molar eminence was comprised of hyaluronic acid (HA) staining in the control embryos (Figure 6A). However, in the mutant embryos we observed a dramatic decrease in HA (Figure 6B). We also observed a dramatic decrease in CD44, an HA receptor, in the same region (Figure 6 C, D). These data suggested that the Irf6 expressing, *Myf5*+ cells surrounding the inter-molar eminence were non-cell-autonomously acting on the CNC cells leading to HA and CD44 production in the posterior tongue.

### Increased Bmp2 and Decreased Bmp4, Shh, and Fgf10 in *Irf6*−/− Tongue

**Figure 7:**
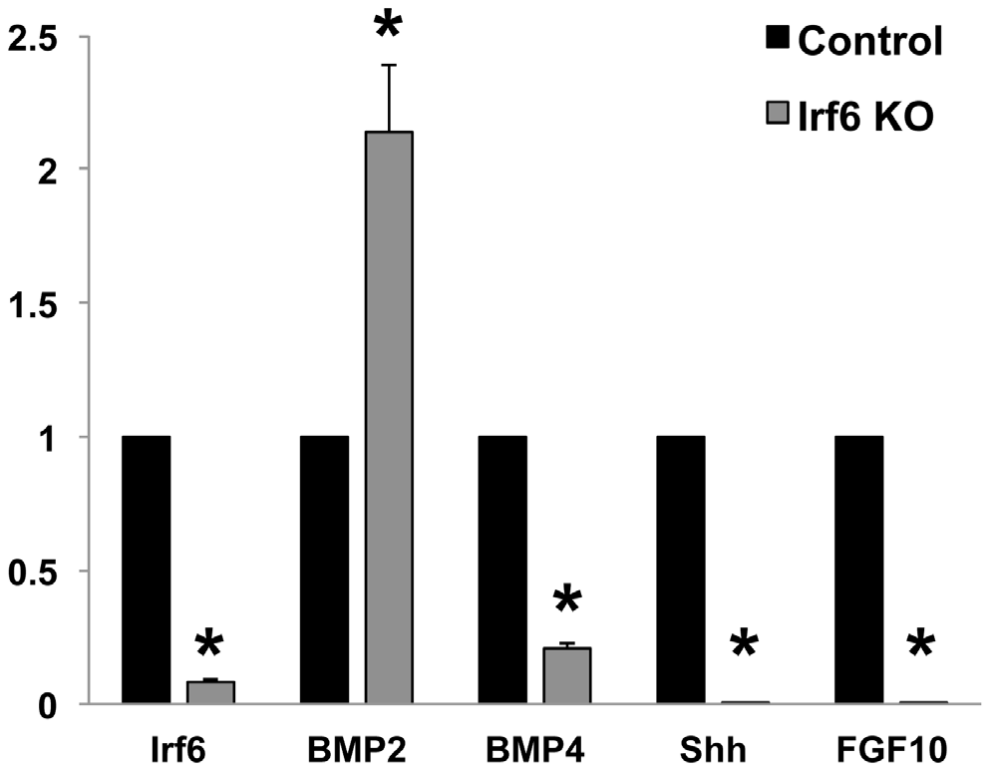
Reduced *Shh, Bmp4* and *Fgf10* in the *Irf6*−/− Tongue. Quantitative PCR of *Irf6*−/− tongue tissue was performed on E14.5 mice and compared to controls. The Irf6ko mice showed relative expression changes where *Shh*, *Bmp4*, and *Fgf10* were greatly reduced whereas *Bmp2* was increased two fold. Columns, median fold change obtained from 5 separate experiments; bars, SEM. * = p<0.05.

To evaluate potential mediators of *Irf6* function from the epithelium and/or SPM on the adjacent CNC cells, we performed triplicate quantitative PCR examining expression of *Bmp2*, *Bmp4*, *Fgf10*, and *Shh* in E14.5 *Irf6*+/+ versus *Irf6*−/− mice (Figure 7). In the tongue, we observed a sharp decrease of *Bmp4*, *Fgf10*, and *Shh* in the *Irf6*−/− lingual tissue compared to the controls (Figure 7 A). Interestingly there was a two-fold increase in *Bmp2* expression in the *Irf6*−/− lingual tissue. Previously, *Bmp2*, *Bmp4*, and *Shh* were shown to be expressed primarily in the developing lingual epithelium from E10.5 to E15.5, concentrated in the developing taste buds. [17], [18]. *Fgf10* expression in the tongue was shown to be expressed in the lingual mesenchyme [19]. These data suggest that *Irf6* was required, for lingual mesenchymal expression of *Fgf10* and that *Irf6* in the lingual epithelium was required for *Shh,* and *Bmp4* expression. It remains unclear why *Bmp2* was upregulated, but may suggest that *Bmp2* is upstream of *Irf6*. Since *Fgf10* was expressed in CNC cells, the loss of this expression in *Irf6* knockout embryos was most likely due to a non-cell-autonomous pathway [19].

## Discussion

### *Irf6* in the Tongue

A great degree of interest has been focused on the role of *Irf6* during epithelial development. Examination of the *Irf6*−/− mutant mice demonstrated misregulation of epithelial markers *loricrin*, *filaggrin*, *p63* and *K14* that led to adhesions in the limb, oral cavity, and the esophagus [2], [3]. Recently Fakhouri et al (2012) demonstrated that *MCS9.7*, an enhancer of *Irf6* expression, was active in the epithelium as expected, but in unexpected areas too, including the non-epithelial tissues of the forelimb, hindlimb and the tongue [6]. Thus, we tested the hypotheses that *Irf6* was expressed in the tongue and was required for lingual development.

The expression pattern of Irf6 in the tongue was identical to the *MCS9.7* fate mapping, suggesting that the enhancer *MCS9.7* acts upstream of *Irf6* during lingual mesodermal development. These data suggest that Irf6 was present during lingual muscular differentiation [19]. Molecular analyses suggest that *Irf6* was epistatic to myosin heavy chain expression, but was likely down-stream of *Myf5* and *myogenin* functions. In *Myf5*-*Cre*;*Smad4 F/F* mice, microglossia occurred due to aberrant myotubule formation and reduced myogenic differentiation. Since these mice had reduced lingual myogenin staining, then *Smad4* is likely to be epistatic to *Irf6* [20]. Although no obvious muscular phenotype has been reported in VWS and PPS patients, myogenic deficiencies have been noted in the orbicularis oris muscle of family members of cleft lip and palate patients, and is consistent with the hypothesis that subtle muscular phenotypes in *IRF6*-related orofacial clefting disorders can be due to aberrant myogenic formation[21]–[23].

### Loss of Lingual Mesodermal *Irf6* expression Results in Cell-Autonomous and Non-Cell Autonomous Defects

During lingual development cells derived from the SPM and CNC migrate into the lingual primordium to create the musculature and adjacent mesenchymal tissues, respectively [14]. Conditional deletion of *Tgfbr2* from the CNC using *Wnt1-Cre;Tgfbr2 F/F* mice demonstrated that *Tgfbr2* acts non-cell-autonomously on the adjacent SPM to induce proliferation via *Fgf10* expression [19]. In a similar fashion, our data suggested that *Irf6* acted upon the adjacent CNC non-cell-autonomously, inducing the inter-molar eminence growth, that included HA and CD44. The origin of the inter-molar eminence was thought to be a remnant of the second branchial arch from median lingual buds, and we demonstrated that this region was comprised of CNC cells [24]. The CNC cells of the inter-molar eminence were surrounded by *Myf5*+;*Irf6*+ SPM cells, and were absent in the *Irf6*−/− embryos. This suggests that loss of *Irf6* in SPM cells of the tongue prevented the normal expansion of the CNC in the posterior tongue. In the *Wnt1-Cre;Tgfbr2 F/F* mice the CNC cells were found to be critical to guiding proper SPM migration and organization, suggesting interdependence between these cell lineages. Both HA and CD44 are known to be involved in organogenesis, and mutation of genes in the HA pathway led to multiple developmental defects [25]. The role that *Irf6* plays during the development of the inter-molar eminence is unknown, but our data strongly suggested that *Irf6* was involved in non-cell-autonomous signaling to the CNC in this area of the tongue.

The role of *Irf6* during development is largely unknown, however its role during epidermal and mammary formation highlights its effect on the cell cycle and cellular differentiation [2], [3], [26]. The decrease in proliferation throughout the *Irf6*−/− lingual tissue suggested that (1) *Irf6* was acting directly on the SPM in a cell-autonomous fashion to affect cell cycle progression and/or (2) acting in a non-cell-autonomous fashion on the CNC, which then may signal back to the SPM, similar to the results seen in the *Wnt1-cre;Tgfbr2 F/F* mice. The *Wnt1-cre;Tgfbr2 F/F* mice had defects in lingual mesodermal proliferation that were non-cell-autonomous [19]. During myogenic differentiation of the lingual SPM, *Irf6* was required in a cell-autonomous role for proper patterning of the actin-myosin cytoskeleton. Actin and myosin comprise the cytoskeleton of the myofibril, following myoblastic determination and differentiation. Abnormalities in actin stabilization and polymerization have been associated with facial developmental defects resulting from aberrant cell adhesion and migration [27]. Comparatively, lingual muscular differentiation in the *Wnt1-cre;Tgfbr2 F/F* and *Wnt1-cre;Hand2 F/F* mutants had normal myosin heavy chain, and normal actin and myosin heavy chain, respectively [19], [28]. *Myf5*-Cre;*Smad4 F/F* mice suffered from abnormal myotubule fusion and reduced myogenin and myosin heavy chain production, which was partially rescued by exogenous *Fgf6* [20]. This suggested that *Irf6* alone was required for terminal lingual muscular differentiation, as evidenced by actin and myosin heavy chain formation. However, while *Tgfbr2* and *Hand2* were required for SPM proliferation and patterning, respectively, they do not determine SPM muscular differentiation, as evidenced by normal actin and myosin formation. *Smad4* in the SPM was found to be necessary for myotubule fusion and myogenic differentiation, suggesting that this pathway may be epistatic to the *Irf6* pathway [20]. Based on these data we cannot tell whether similarities of the reduced SPM tongue phenotype in the *Irf6*−/− and *Wnt1-Cre;Tgfbr2*−/− are due to common or parallel pathways.

### Mechanism(s) of *Irf6* Function in the Tongue

The mechanism(s) of *Irf6’s* role in the tongue that led to altered SPM migration, aberrant cytoskeletal formation, reduced proliferation and absent CNC-derived HA/CD44 production was unknown. We used quantitative PCR and MALDI-TOF to evaluate alterations in the gene expression patterns and protein profile of *Irf6*−/− tongue for clues to explain these findings.

Multiple growth factors are expressed during tongue formation although the majority of research has focused on their role during epithelial development. Sonic Hedgehog (*Shh*) is a critical soluble growth factor required for tongue development. *Myf5* is a long-range target of *Shh* and is necessary for hypaxial somatic migration and is a marker of tongue myogenic determination [29], [30]. *Shh* is expressed in the lingual and palatal epithelium and *Shh* has been found in the palate to belong to a signaling cascade involving *Fgf10, Bmp2* and *Bmp*4 [31]–[34]. *Bmp2* is expressed in the developing lingual epithelium, and is concentrated in the forming taste buds [18]. The role of *Shh* in papilla and taste bud development has been well characterized and early blockade of *Shh* leads to tongue agenesis [17], [35], [36]. The presence of *Patched1*, a receptor for *Shh*, in the CNC of the tongue suggests that tongue development requires *Shh*. Deletion of *Smoothened* from the CNC using Wnt1-cre;*SmoF/F* mice led to severe craniofacial defects including paired hypoplastic tongue buds with absent *Myf5* expression. During papilla and taste bud formation, epithelially expressed *Shh* acts upon the underlying lingual mesenchyme, where Smoothened, and down stream target *Gli* are active and expressed [18], [35], [37]. In the *Irf6*−/− mice there was significant down-regulation of *Shh,* and *Bmp4* in the lingual tissue suggesting that loss of *Irf6* signaling was required for *Shh* and *Bmp4* expression via a cell-autonomous role. Upregulation of *Bmp2* in the *Irf6*−/−lingual tissue suggests that *Bmp2* may act upstream of *Irf6* in the lingual epithelium.

Similarly we found a global reduction of *Fgf10* in the *Irf6*−/− tongue tissue. These data suggest that *Fgf10* was acting in a signaling pathway where *Irf6* expression in the overlying epithelium or the adjacent SPM non-cell-autonomously activates *Fgf10* expression in the adjacent CNC. In *Fgf10*−/− mice, there was reduced cellular proliferation in the palate, similar to that seen in the *Irf6*−/− and *Wnt1-Cre;TgfbR2 F/F* tongue suggesting that *Fgf10* was necessary for SPM proliferation.

*In situ* evaluation of the proteomes from the *Irf6*−/− lingual tissue reported significant alterations at the proteomic level using MALDI IMS. We topographically identified increased Thymosin B4 and B10 in the lingual mesenchyme. Thymosin B4 (TB4) and B10 (TB10) are small polypeptides with multiple roles during development including angiogenesis, wound repair and cytoskeletal formation. Most notably, TB4 and TB10 are often found to be co-expressed and actively bind to G-actin, preventing it from polymerizing (reviewed in Smart) [38]. The increase of TB4 and TB10 may effect the ability of the *Myf5*+ cells to migrate and differentiate in the *Irf6*−/− tongue in addition to effecting actin polymerization. This suggests that Irf6 suppresses TB4 and TB10 in the SPM cells to promote actin polymerization and myogenic differentiation via myosin production. Conversely, the *Wnt1-Cre;Tgfbr2 F/F* mice have reduced SPM due to reduced proliferation but normal myosin production [19]. These findings suggest that the *Wnt1-Cre;Tgfbr2 F/F* phenotype was related to reduced *Fgf10* expression from the CNC, whereas the *Irf6*−/− myogenic phenotype was more likely related to a cell-autonomous function of *Irf6* in the SPM. It also remains possible that the effects observed in the SPM of the *Irf6*−/− tongue are due to altered signaling between the SPM and CNC via soluble growth factors. Certainly the CNC phenotype in the *Irf6*−/− inter-molar eminence suggest that soluble mediator(s) of *Irf6* function must exist for this non-cell-autonomous function to occur, as noted in the Wnt1-Cre;*Tgfbr2 F/F* mice.

The ultimate phenotype present in the *Irf6*−/− tongue was aberrant extracellular and cytoskeletal formation associated with a reduced *Myf5*+ cell population. It remains to be determined which growth factors are responsible for the alterations in BT4 and BT10 in the *Irf6*−/− tongue. The degree to which epithelial loss of *Irf6* effected adjacent lingual development was unknown but will be studied in the near future using a conditional *Irf6* allele. The aberrant formation of the cytoskeletal elements and reduced cell proliferative capacity in the *Irf6*−/− mice would likely lead to poor tissue strength and decreased wound repair if these mice survived. These findings may explain why humans with *IRF6* mutations exhibit increased wound healing complications after cleft lip and palate repair [39].

## Supporting Information

Figure S1 **Co-Labeling of** ***Wnt1-Cre*** **and** ***Irf6*** **in Tongue.** Coronal sections of E15.5 tongue of *Wnt1-Cre;R26R* control embryo co-labeled with *Irf6* (green) and B-Galatosidase (red) staining. At 10x *Irf6* staining was seen throughout the SPM area of the tongue (A, D, E) whereas B-Gal activity was shown to be adjacent to these striations of the transversal muscle (B, E, H). Higher magnification of the tongue (circle in A, B, C) at 20x (D, E, F) and 40x (G, H, I) revealed that *Irf6* was expressed adjacent to, but not within the Wnt1+ cells, indicating that *Irf6* was not expressed primarily in the CNC.
(TIF)

Figure S2 **Lineage tracing of the CNC using** ***Wnt1-Cre;R262R*** **in** ***Irf6*** **−/− Embryos.** Coronal sections through anterior, middle and posterior tongue of control and *Irf6*−/− mice at E13.5, E14.5 and E15.5. In the E13.5 control mice there was predominant CNC contribution (blue staining) to the tongue (A, C, E) except for striations of the transversal muscle seen in body of the tongue extending from the midline (white arrow). In the E13.5 *Irf6*−/− tongues there was a relative increase of CNC (blue cells in B, D, F) noted with relative reduction of non-CNC cells (pink cells) in the striated portions of the tongue of the transversal muscle (white arrow). At E14.5 there was again strong contribution of CNC to the control tongue (blue cells in G, I, K) however there was an increasing contribution of non-CNC cells in the tongue (pink cells) especially posteriorly. In the E14.5 *Irf6*−/− mice there was a relative increase in CNC cells (blue cells in H, J, I) and a reduction in non-CNC (pink cells) throughout the tongue. At E 15.5 controls there was a reduction of CNC contribution to the tongue (blue cells in M, O, Q) compared to non-CNC (pink cells) especially in the posterior tongue (white arrow). In the E15.5 *Irf6*−/− tongue’s maintain a relative predominance of CNC (blue cells in N, P, R) with a decrease in non-CNC (pink cells) especially in the posterior tongue (white arrows). The CNC also contributes to the inter-molar eminence seen in control tongues but absent in the *Irf6*−/− (white asterisk).
(TIF)

Figure S3 **Co-localization of PhosphoHistone H3 and** ***Myf5*** **in** ***Irf6*** **−/− Tongue.** Coronal sections through E13.5, E14.5 and E15.5 anterior and posterior tongue tissue from control and *Irf6*−/− mice. At E13.5, E14.5, and E15.5 there is qualitatively more PH3+ cells (green) that co-localize with B-Gal staining (red), representing the segmental paraxial mesenchyme. In the E13.5, E14.5, and E15.5 *Irf6*−/− mice there was a qualitative global reduction in PH3+ cells in the B-Gal+and B-Gal- cells, similar to Figure 3.
(TIF)

Figure S4 **Immunohistochemistry of** ***Thymosin B4*** **and** ***B10*** **in** ***Irf6*** **−/− Tongue.** Coronal sections through control and *Irf6*−/− tongue tissue. Using immunohistochemistry we identified increased *BT4* (B) and *BT10* (D) expression in the *Irf6*−/− tongue compared to the controls (C, D respectively).
(TIF)
