# Supplementary figures and images for: Cell-Autonomous and Non-Cell-Autonomous Roles for Irf6 during Development of the Tongue
Source: PLoS One. 2013 Feb 22;8(2):e56270. doi: 10.1371/journal.pone.0056270 (PMC3579850; doi:10.1371/journal.pone.0056270)

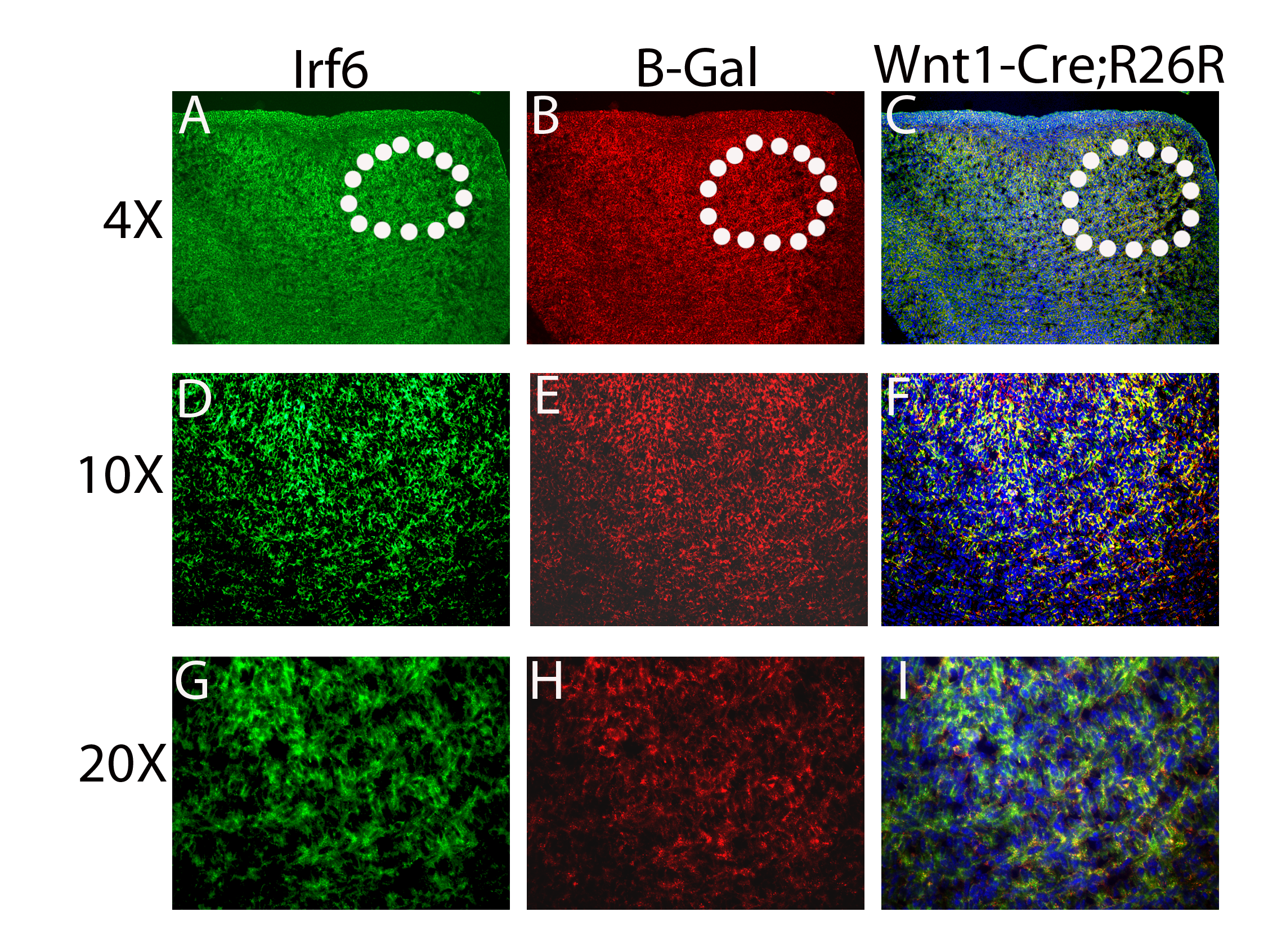

Supplement: Figure S1 — Co-Labeling of Wnt1-Cre and Irf6 in Tongue. Coronal sections of E15.5 tongue of Wnt1-Cre;R26R control embryo co-labeled with Irf6 (green) and B-Galatosidase (red) staining. At 10x Irf6 staining was seen throughout the SPM area of the tongue (A, D, E) whereas B-Gal activity was shown to be adjacent to these striations of the transversal muscle (B, E, H). Higher magnification of the tongue (circle in A, B, C) at 20x (D, E, F) and 40x (G, H, I) revealed that Irf6 was expressed adjacent to, but not within the Wnt1+ cells, indicating that Irf6 was not expressed primarily in the CNC. (TIF) [file pone.0056270.s001.tif]

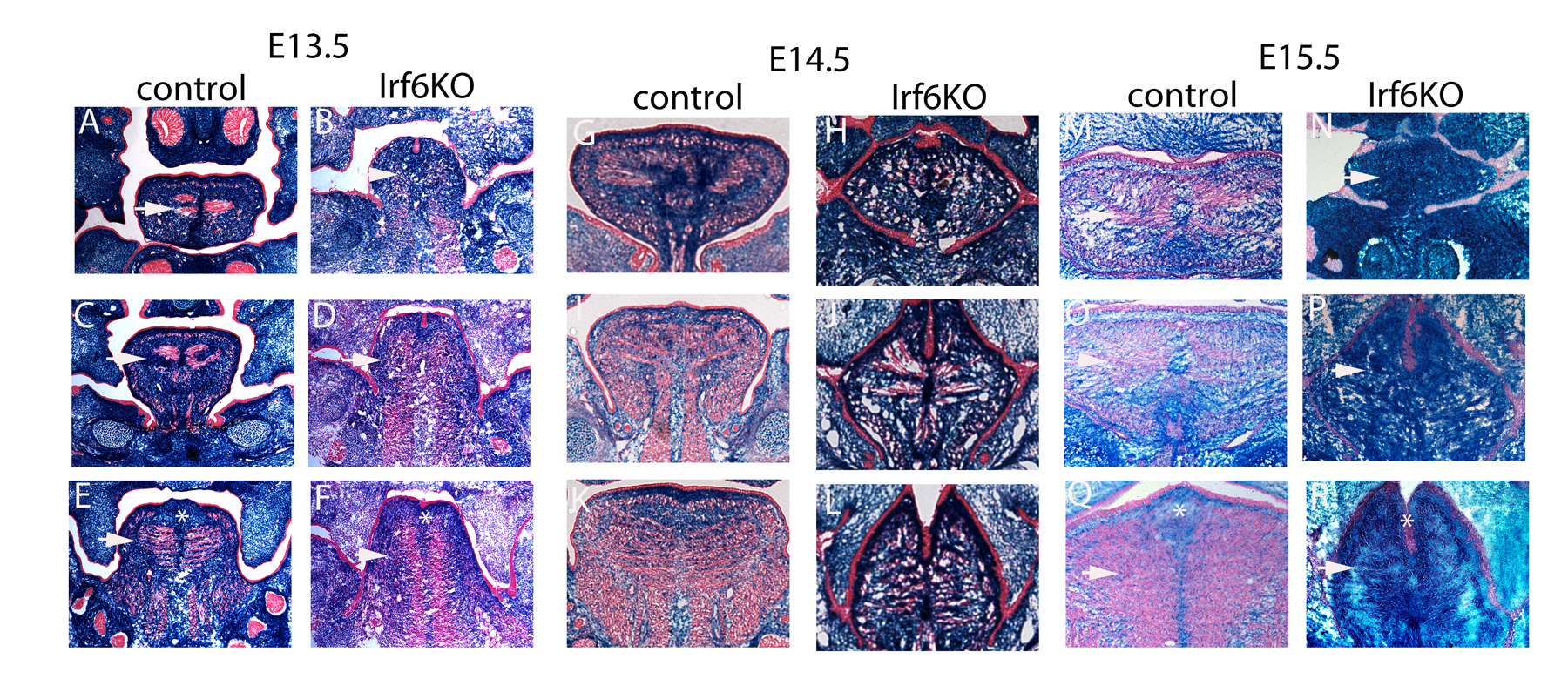

Supplement: Figure S2 — Lineage tracing of the CNC using Wnt1-Cre;R262R in Irf6 −/− Embryos. Coronal sections through anterior, middle and posterior tongue of control and Irf6−/− mice at E13.5, E14.5 and E15.5. In the E13.5 control mice there was predominant CNC contribution (blue staining) to the tongue (A, C, E) except for striations of the transversal muscle seen in body of the tongue extending from the midline (white arrow). In the E13.5 Irf6−/− tongues there was a relative increase of CNC (blue cells in B, D, F) noted with relative reduction of non-CNC cells (pink cells) in the striated portions of the tongue of the transversal muscle (white arrow). At E14.5 there was again strong contribution of CNC to the control tongue (blue cells in G, I, K) however there was an increasing contribution of non-CNC cells in the tongue (pink cells) especially posteriorly. In the E14.5 Irf6−/− mice there was a relative increase in CNC cells (blue cells in H, J, I) and a reduction in non-CNC (pink cells) throughout the tongue. At E 15.5 controls there was a reduction of CNC contribution to the tongue (blue cells in M, O, Q) compared to non-CNC (pink cells) especially in the posterior tongue (white arrow). In the E15.5 Irf6−/− tongue’s maintain a relative predominance of CNC (blue cells in N, P, R) with a decrease in non-CNC (pink cells) especially in the posterior tongue (white arrows). The CNC also contributes to the inter-molar eminence seen in control tongues but absent in the Irf6−/− (white asterisk). (TIF) [file pone.0056270.s002.tif]

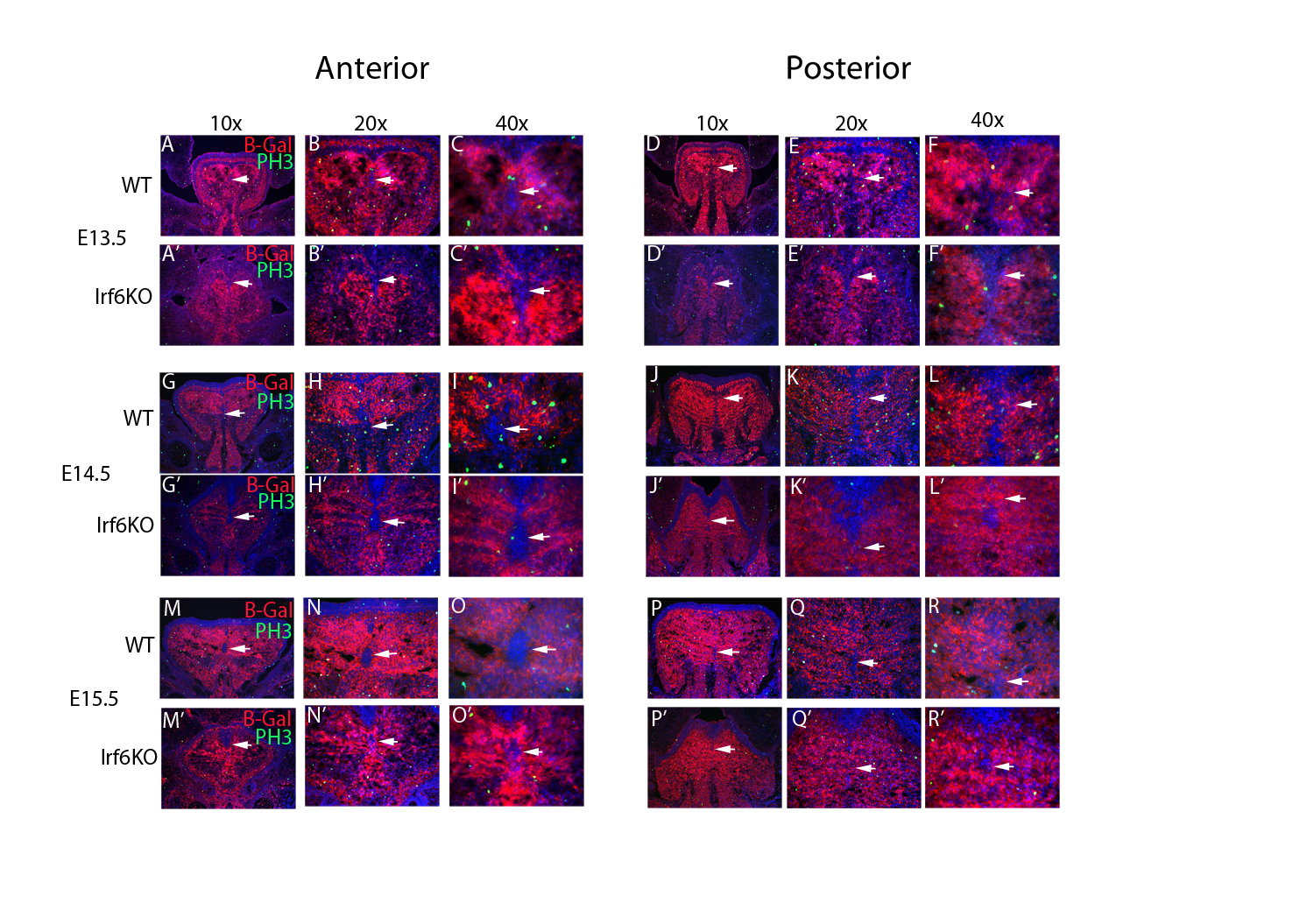

Supplement: Figure S3 — Co-localization of PhosphoHistone H3 and Myf5 in Irf6 −/− Tongue. Coronal sections through E13.5, E14.5 and E15.5 anterior and posterior tongue tissue from control and Irf6−/− mice. At E13.5, E14.5, and E15.5 there is qualitatively more PH3+ cells (green) that co-localize with B-Gal staining (red), representing the segmental paraxial mesenchyme. In the E13.5, E14.5, and E15.5 Irf6−/− mice there was a qualitative global reduction in PH3+ cells in the B-Gal+and B-Gal- cells, similar to Figure 3. (TIF) [file pone.0056270.s003.tif]

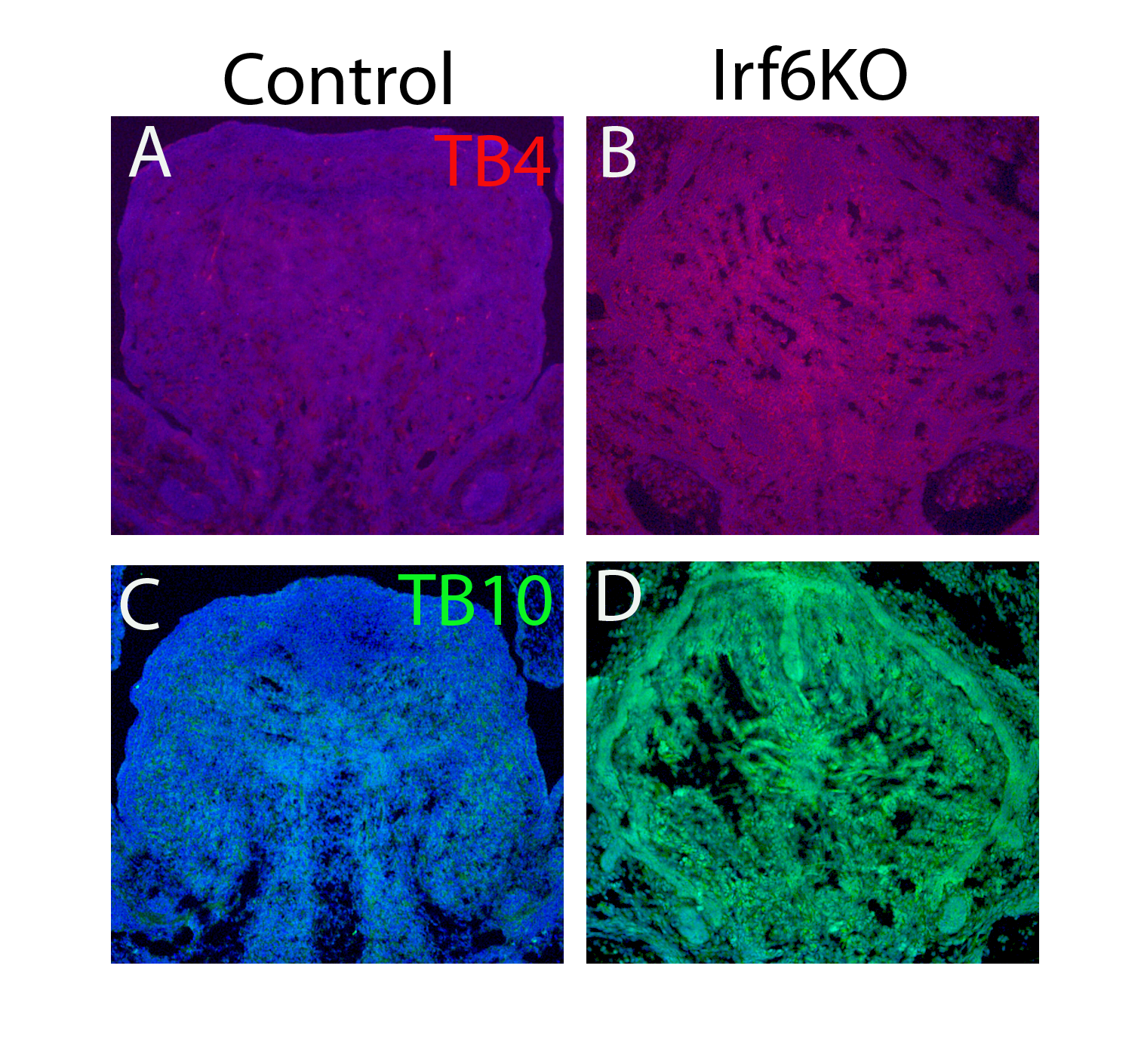

Supplement: Figure S4 — Immunohistochemistry of Thymosin B4 and B10 in Irf6 −/− Tongue. Coronal sections through control and Irf6−/− tongue tissue. Using immunohistochemistry we identified increased BT4 (B) and BT10 (D) expression in the Irf6−/− tongue compared to the controls (C, D respectively). (TIF) [file pone.0056270.s004.tif]
